# Time-of-Day Effects on Competitive Speedcubing Performance

**DOI:** 10.5334/jcr.266

**Published:** 2026-05-25

**Authors:** Sanjay Adireddi Adireddi

**Affiliations:** 1 Ladue Horton Watkins High School, United States

**Keywords:** time of day, speedcubing, post-prandial alertness dip, circadian rhythm, cognitive-motor performance

## Abstract

Competitive speedcubing — the timed solving of Rubik’s Cube puzzles under standardised World Cube Association (WCA) rules — uniquely couples rapid visuospatial processing and working memory with high-speed bimanual fine motor execution, making it a tractable paradigm for investigating time-of-day associations in real-world expert cognitive-motor performance. We analysed 6,600 record-setting performances across four puzzle categories (3 × 3 × 3, 4 × 4 × 4, 5 × 5 × 5, 6 × 6 × 6) from WCA competitions held between 2003 and 2025 using an exposure-adjusted framework in which record probability is expressed as records per attempt within 15-minute local-time bins, evaluated against a within-competition permutation null that preserves schedule structure. Raw record counts exhibit a bimodal distribution with morning and evening peaks; however, exposure adjustment demonstrates that this pattern is substantially attributable to the non-uniform scheduling of competition rounds across the day. After schedule correction, the 4 × 4 × 4 event shows a statistically significant suppression of record probability during the 12:30–15:00 window (outside-to-inside ratio: 1.14×; one-tailed permutation *p* = 0.020), consistent in timing with the post-prandial alertness dip. This effect is not statistically significant for the 3 × 3 × 3, 5 × 5 × 5, or 6 × 6 × 6 events. Sensitivity analyses confirm that the 4 × 4 × 4 finding is broadly distributed across regions and competitive eras and persists within early rounds alone. The late-evening elevation in raw counts is explained by final-round competitor selection. These results demonstrate the methodological importance of exposure adjustment in ecological performance analyses and identify a residual midday suppression in 4 × 4 × 4 record probability that warrants further investigation.

## Introduction

The suprachiasmatic nucleus (SCN) of the hypothalamus generates endogenous circadian rhythms propagated to peripheral tissues and cortical regions partly through the timed release of glucocorticoids [1]. These rhythms coordinate daily fluctuations in arousal, sustained attention, executive function, and motor excitability [2,3]. Attentional performance typically improves across the morning, while a transient reduction in alertness — the post-prandial dip — occurs between approximately 12:30 and 15:00, independent of meal ingestion and linked to the 12-hour harmonic of the circadian system [4,5]. The SCN-driven circuit underlying these effects includes vasoactive intestinal peptide (VIP)-producing neurons that entrain paraventricular nucleus rhythms to time the daily release of glucocorticoids [1], which in turn modulate cortical excitability.

Competitive speedcubing offers a well-powered naturalistic setting for studying time-of-day associations in expert performance. A defining feature of speedcubing, and a key distinction from paradigmatically cognitive competitive tasks such as chess, is that every solve couples rapid visuospatial analysis, algorithmic working memory, and executive decision-making with high-speed bimanual fine motor execution. Because fine motor performance and cognitive performance exhibit distinct circadian profiles [6], this concurrent cognitive-motor demand may produce time-of-day sensitivity not observed in purely cognitive tasks [7]. Record-setting performances (world (WR), continental (CR), and national (NR) records) are elite-gated events: a record can only be set by a competitor whose performance surpasses all prior verified performances in their jurisdiction, meaning low-skill attempts cannot enter the numerator and do not confound record counts as a signal of elite performance. Larger puzzles (4 × 4 × 4–6 × 6 × 6) require proportionally more planning per solve, providing a natural cognitive load gradient within the same framework.

We report an exposure-adjusted analysis of time-of-day variation in WCA record-setting probability across four puzzle categories, with record probability expressed as records per attempt in 15-minute local-time bins evaluated against a within-competition permutation null. The Fewest Moves Challenge was excluded due to insufficient sample size (*n* = 41 records).

## Methods

### Data sources and WCIF coverage

The full WCA public export (version: May 2025) was downloaded, comprising results from 17,092 competitions held between 2003 and 2025. Of these, 14,108 held 3 × 3 × 3 events and were submitted to schedule retrieval via the WCA Competition Information Format (WCIF) public API. Of the 14,108 queried, 9,259 (65.6%) had machine-readable round start times and were retained. The remaining 4,849 were excluded: 4,829 because their WCIF files contain no venue or activity records, as these competitions predate the WCA’s adoption of electronic scheduling (principally 2003–2012); 14 because no JSON file was available; and 6 for other parseable failures. All 9,259 retained competitions had valid IANA timezone strings; no geo-imputation was required.

### Record inclusion and pooling

WR, CR, and NR records were pooled across single and average categories to maximise statistical power; the per-event WR/CR/NR breakdown is reported in figure panel labels. Records were pooled across tiers because all three share the same elite-gating property and per-tier stratification would substantially reduce per-bin sample sizes. Era effects are captured implicitly by the exposure denominator, which scales with attempt volume in each bin.

### Local time derivation

Round start times were extracted from WCIF venue records as UTC timestamps and converted to local civil time using the IANA timezone database (Python *pytz* library, version ≥2023.3). Daylight saving time transitions were handled automatically. Within each competition, the (competitionId, eventId, round_number) tuple was mapped to a WCA roundTypeId using the rank-ordered sequence of round types contested. The term ‘round start local time’ refers consistently to the local civil time of the scheduled round start, not the time of any individual solve attempt.

### Exposure-adjusted record probability

Local round start times were discretised into 15-minute bins. For each bin *t*, record probability was computed as:


P(record | t) = (records in rounds starting in bin t)/                                           (total attempts in rounds starting in bin t)

Rates are displayed as records per *N* attempts, scaled per event (3 × 3 × 3: per 100,000; 4 × 4 × 4: per 50,000; 5 × 5 × 5: per 20,000; 6 × 6 × 6: per 5,000). A minimum-attempts filter excluded bins with insufficient exposure: 10,000 for 3 × 3 × 3; 7,825 for 4 × 4 × 4; 3,954 for 5 × 5 × 5; 977 for 6 × 6 × 6. Poisson 95% confidence intervals are shown as shaded ribbons in Figure 2.

### Permutation null model and test statistics

A within-competition permutation test was implemented (10,000 iterations; NumPy *default_rng*, seed = 42). For each iteration, record labels were shuffled within each competition-event-round stratum via multinomial reallocation proportional to observed attempt counts, preserving total record count and competition-level exposure structure. For overall rate non-uniformity, the test statistic was the normalised maximum absolute deviation:


$$T\;\text{=}\;\text{max}(|r_{ο}\;-\;\bar{r}|)/\bar{r}$$

where $r_{ο}$ is the rate at bin *b* and $\bar{r}$ is the mean rate across valid bins. For the post-prandial window comparison (12:30–15:00), the test statistic was the outside-to-inside rate ratio. In both the observed and all permuted calculations, the outside-window rate was computed over all bins with at least one attempt, ensuring identical bin sets across observed and null statistics. All reported *p*-values are one-tailed.

### Sensitivity analyses

For the 4 × 4 × 4 event, the outside-to-inside dip ratio was computed separately by continent, competitive era (pre-2023: 2011–2022; 2023+: 2023–2025), and round stage (early rounds 1–2 versus finals), using the same rate metric (records per 50,000 attempts) and window definition throughout.

## Results

### Raw record count distributions

**Figure 1:**
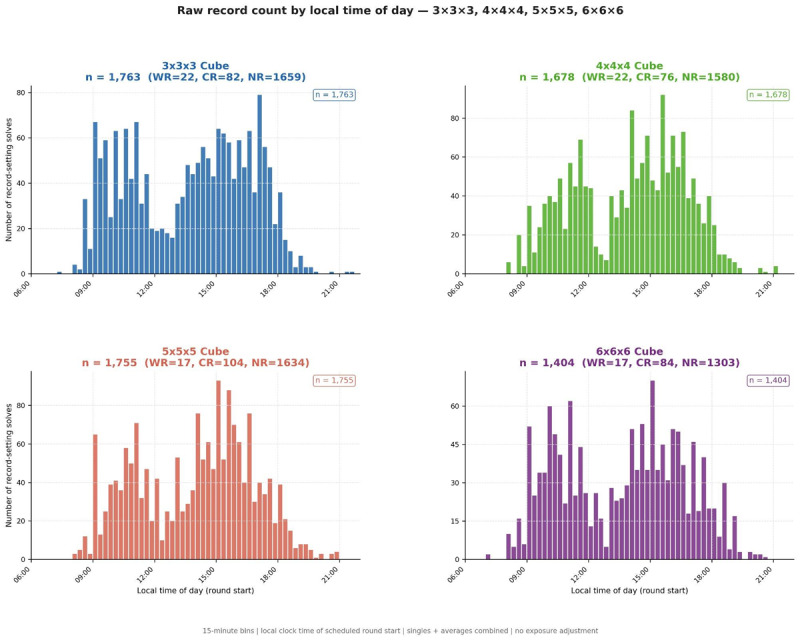
Raw record-setting solve counts by local time of day for all four events. Panels show 3 × 3 × 3 (blue, n = 1,763), 4 × 4 × 4 (green, n = 1,678), 5 × 5 × 5 (red, n = 1,755), and 6 × 6 × 6 (purple, n = 1,404). Bars represent counts in 15-minute bins by local civil time of scheduled round start. WR/CR/NR breakdown shown in panel titles. No exposure adjustment applied.

Figure 1 presents raw record counts in 15-minute bins for all four events (total *n* = 6,600: 3 × 3 × 3, *n* = 1,763 [WR = 22, CR = 82, NR = 1,659]; 4 × 4 × 4, *n* = 1,678 [WR = 22, CR = 76, NR = 1,580]; 5 × 5 × 5, *n* = 1,755 [WR = 17, CR = 104, NR = 1,634]; 6 × 6 × 6, *n* = 1,404 [WR = 17, CR = 84, NR = 1,303]). All four events show a bimodal distribution: a morning concentration peaking between approximately 09:00 and 11:00, a midday reduction, and a second rise peaking between 17:00 and 18:30. Because WCA competitions schedule a disproportionate number of rounds in morning hours, this raw distribution reflects combined scheduling and performance influences and cannot be interpreted as evidence of time-of-day variation in record probability without exposure adjustment.

### Exposure-adjusted record probability

**Figure 2:**
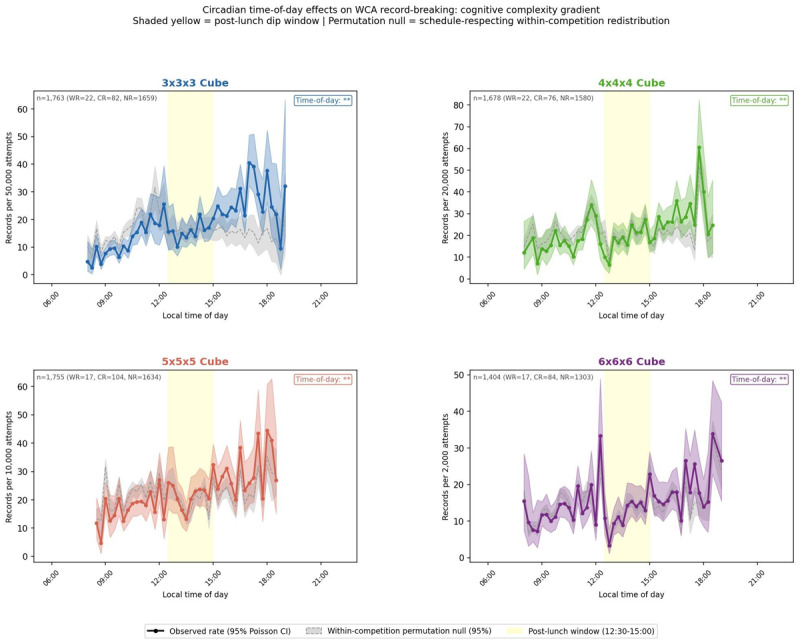
Exposure-adjusted record probability by local time of day (10,000-iteration within-competition permutation null). Solid lines show observed rates (records per N attempts) with 95% Poisson confidence intervals (shaded ribbons). Gray dashed bands show the 95% permutation null interval. Yellow shading marks the post-prandial window (12:30–15:00). **p < 0.05, one-tailed permutation test. n and WR/CR/NR breakdown in panel titles. Minimum-attempts thresholds: 3 × 3 × 3, 10,000; 4 × 4 × 4, 7,825; 5 × 5 × 5, 3,954; 6 × 6 × 6, 977.

After exposure adjustment (Figure 2), apparent morning enrichment is substantially attenuated across all four events. Overall non-uniformity does not reach significance for any event (3 × 3 × 3: *p* = 0.175; 4 × 4 × 4: *p* = 0.254; 5 × 5 × 5: *p* = 0.053; 6 × 6 × 6: *p* = 0.065). For the post-prandial window, the 4 × 4 × 4 event shows a statistically significant suppression (inside: 26.51 per 50,000 attempts; outside: 30.20; ratio: 1.14×; *p* = 0.020). This suppression is consistent in timing with the post-prandial alertness dip [4,5] and is treated as a plausible alignment rather than confirmatory evidence of circadian modulation. The comparison does not reach significance for 3 × 3 × 3 (inside: 18.64 per 100,000; outside: 17.65; ratio: 0.95×; *p* = 0.385), 5 × 5 × 5 (inside: 22.32 per 20,000; outside: 24.20; ratio: 1.08×; *p* = 0.858), or 6 × 6 × 6 (inside: 16.53 per 5,000; outside: 19.79; ratio: 1.20×; *p* = 0.493). For 3 × 3 × 3, the rate is marginally higher inside the window, directionally inconsistent with a post-prandial suppression. Null results should not be interpreted as evidence of absence; power is constrained by the WCIF-matched sample.

### Sensitivity analyses: 4 × 4 × 4 post-prandial effect

The 4 × 4 × 4 dip ratio is broadly distributed across regions. Asia (*n* = 214; ratio: 1.31×) and the Americas (*n* = 154; ratio: 1.17×) both show effects consistent with the overall direction. Europe, the largest contributor (*n* = 466; ratio: 1.00×), modestly dilutes the aggregate but does not drive it. Oceania (*n* = 35) is underpowered for inference. The effect is consistent across eras (pre-2023: 1.11×; 2023+: 1.21×), indicating no dependence on any particular phase of WCA competition growth. Critically, the dip persists within early rounds alone (rounds 1–2: ratio 1.20×, *n* = 421), ruling out final-round scheduling as a necessary driver. Finals show a marginal ratio of 1.04× (*n* = 493), consistent with finals being held predominantly in the evening regardless of time of day.

### Evening elevation and final-round composition

At the peak evening bin, final rounds accounted for 79.2% of 3 × 3 × 3 records (peak at 18:00), 65.0% of 4 × 4 × 4 (17:45), 50.0% of 5 × 5 × 5 (18:00), and 100% of 6 × 6 × 6 (18:30). The evening elevation in raw counts is therefore attributable to competitor selection and scheduling conventions rather than time-of-day performance enhancement.

## Discussion

### Principal findings

Exposure adjustment demonstrates that non-uniform round scheduling is the dominant driver of apparent morning enrichment in raw WCA record counts. After schedule correction, only the 4 × 4 × 4 event shows a residual midday suppression surviving permutation testing. The directional consistency across events and the power limitations of the WCIF-matched sample preclude strong conclusions about absence of effect in the remaining events. The late-evening spike is explained by final-round competitor selection, supported by the round-stage composition data.

### The cognitive-motor profile of speedcubing and ecological comparators

The concurrent cognitive-motor demand of speedcubing is central to interpreting any time-of-day associations. Every solve requires not only rapid visuospatial analysis and executive sequencing but also the concurrent execution of high-speed bimanual fine motor movements. Sleep quality and circadian regularity have been examined in competitive chess [7], a task isolating the cognitive dimension without concurrent motor demand. The additional fine motor execution component may produce a distinct circadian sensitivity profile, since neuromuscular parameters including reaction time, manual dexterity, and motor cortex excitability exhibit time-of-day variation that does not necessarily align with the cognitive performance curve [6]. Whether the combined cognitive-motor demand amplifies, attenuates, or shifts the timing of time-of-day effects relative to purely cognitive tasks is an open empirical question.

### Post-prandial alertness dip

The suppression of 4 × 4 × 4 record probability during 12:30–15:00 aligns in timing with the post-prandial dip, a transient alertness reduction linked to the 12-hour harmonic of the circadian system [4,5]. Consistent with a biological origin, the afternoon alertness reduction is documented across industrialised and non-industrialised societies alike, and cross-cultural actigraphy studies of pre-industrial populations including the Hadza of Tanzania, the San of Namibia, and the Tsimane of Bolivia reveal consistent circadian sleep-wake organisation across genetically and geographically distinct groups, suggesting that the 12-hour harmonic of the circadian system reflects an ancestral feature of human biology [8]. This is treated as a plausible hypothesis: without chronotype data, circadian phase markers, or random assignment of round times, a causal attribution to circadian physiology is unwarranted. The 4 × 4 × 4 solve requires more planning and sustained working memory than the 3 × 3 × 3, consistent with evidence that higher cognitive load may increase circadian sensitivity [2], though this remains speculative.

### Evening elevation: scheduling and motivational arousal

The compositional explanation for the evening spike is most parsimonious. A complementary neurobiological contribution cannot be excluded: the locus coeruleus–norepinephrine system is activated under high motivational salience and enhances neural gain of prioritised representations [9]. In final rounds, where stakes are maximal and each solve is a competitor’s last opportunity, LC-NE-mediated arousal may sharpen visuospatial and motor execution independently of time-of-day effects. Disentangling competitor-selection from arousal-mediated contributions requires round-stage-stratified analyses with chronotype covariates.

### Limitations

This analysis is observational and does not establish causality. Individual chronotype, sleep quality, transmeridional travel, and pre-competition warm-up routines are unobserved and could contribute to apparent time-of-day associations. The WCA competitor base is weighted toward adolescents and young adults, who carry a later average chronotype than older adults [10], making any morning-associated effect potentially conservative. WCIF coverage is limited to competitions with recoverable digital schedule data, underrepresenting earlier events. Records were pooled across tiers and categories to maximise power; stratified analyses and era fixed effects are directions for future work. Local clock time serves as a proxy for circadian phase throughout; individual differences in entrainment mean these quantities are not equivalent, and future work should incorporate direct phase markers.

## Conclusion

Exposure adjustment attenuates apparent morning enrichment in speedcubing record counts, identifying round scheduling concentration as the dominant driver of that pattern. The 4 × 4 × 4 event shows a residual midday suppression consistent with the post-prandial alertness dip that survives schedule correction and is robust across regions, eras, and early rounds in isolation. The late-evening spike is explained by final-round competitor selection. The coupling of rapid cognitive processing with high-speed bimanual fine motor execution distinguishes speedcubing from purely cognitive competitive tasks and warrants dedicated investigation of its time-of-day sensitivity profile. These findings support the value of WCA data as a large-scale naturalistic resource for studying time-of-day associations in performance.

## Data Availability

Competition results were obtained from the publicly available WCA export (https://www.worldcubeassociation.org/export/results). WCIF schedule data were retrieved via the public WCA API. Analysis code is available from the corresponding author upon reasonable request and will be deposited in an open repository upon publication.
